# Impact of the Genome Wide Supported *NRGN* Gene on Anterior Cingulate Morphology in Schizophrenia

**DOI:** 10.1371/journal.pone.0029780

**Published:** 2012-01-12

**Authors:** Kazutaka Ohi, Ryota Hashimoto, Yuka Yasuda, Kiyotaka Nemoto, Takashi Ohnishi, Motoyuki Fukumoto, Hidenaga Yamamori, Satomi Umeda-Yano, Takeya Okada, Masao Iwase, Hiroaki Kazui, Masatoshi Takeda

**Affiliations:** 1 Department of Psychiatry, Osaka University Graduate School of Medicine, Osaka, Japan; 2 National Hospital Organization, Yamato Mental-Medical Center, Nara, Japan; 3 Core Research for Evolutionary Science and Technology of Japan Science and Technology Agency, Saitama, Japan; 4 Molecular Research Center for Children's Mental Development, United Graduate School of Child Development, Osaka University, Kanazawa University and Hamamatsu University School of Medicine, Osaka, Japan; 5 Department of Neuropsychiatry, Institute of Clinical Medicine, University of Tsukuba, Ibaraki, Japan; 6 Department of Psychosomatic Research, National Institute of Mental Health, National Center of Neurology and Psychiatry, Tokyo, Japan; 7 CNS Science Department, Scientific Affairs Division, Janssen Pharmaceutical K.K., Tokyo, Japan; 8 Department of Molecular Neuropsychiatry, Osaka University Graduate School of Medicine, Osaka, Japan; Department of Psychiatry, Japan

## Abstract

**Background:**

The rs12807809 single-nucleotide polymorphism in *NRGN* is a genetic risk variant with genome-wide significance for schizophrenia. The frequency of the T allele of rs12807809 is higher in individuals with schizophrenia than in those without the disorder. Reduced immunoreactivity of *NRGN*, which is expressed exclusively in the brain, has been observed in Brodmann areas (BA) 9 and 32 of the prefrontal cortex in postmortem brains from patients with schizophrenia compared with those in controls.

**Methods:**

Genotype effects of rs12807809 were investigated on gray matter (GM) and white matter (WM) volumes using magnetic resonance imaging (MRI) with a voxel-based morphometry (VBM) technique in a sample of 99 Japanese patients with schizophrenia and 263 healthy controls.

**Results:**

Although significant genotype-diagnosis interaction either on GM or WM volume was not observed, there was a trend of genotype-diagnosis interaction on GM volume in the left anterior cingulate cortex (ACC). Thus, the effects of *NRGN* genotype on GM volume of patients with schizophrenia and healthy controls were separately investigated. In patients with schizophrenia, carriers of the risk T allele had a smaller GM volume in the left ACC (BA32) than did carriers of the non-risk C allele. Significant genotype effect on other regions of the GM or WM was not observed for either the patients or controls.

**Conclusions:**

Our findings suggest that the genome-wide associated genetic risk variant in the *NRGN* gene may be related to a small GM volume in the ACC in the left hemisphere in patients with schizophrenia.

## Introduction

Schizophrenia is a common and complex psychiatric disorder that has a strong genetic component; the estimated heritability is 81% [1]. Many genes have been implicated in the pathogenesis of schizophrenia [2].

A genome-wide association study (GWAS) of single-nucleotide polymorphisms (SNPs) conducted by accessing thousands of DNA samples from patients and controls can be a powerful tool for identifying common risk factors for such a complex disease. Stefansson et al. examined a combined sample of 12,945 patients with schizophrenia and 34,591 controls from three large GWASs (the SGENE-plus, the International Schizophrenia Consortium and the Molecular Genetics of Schizophrenia) and a follow-up with 4,999 patients and 15,555 controls from four additional sample sets from various areas of Europe (including the Netherlands, Denmark, Germany, Hungary, Norway, Russia, Sweden, Finland and Spain) [3]. The researchers identified several significant association signals. Seven markers gave *p* values smaller than the genome-wide significance threshold of approximately 1.6×10^−7^ in the combined samples. Five of these markers—rs6913660, rs13219354, rs6932590, rs13211507 and rs3131296—span the major histocompatibility complex (MHC) region on chromosome 6p21.3–22.1; one marker, rs12807809, is located 3,457 bases upstream from the neurogranin (*NRGN*) gene on 11q24.2; one additional marker, rs9960767, is located in intron four of the transcription factor 4 (*TCF4*) gene on 18q21.2. Of these seven SNPs, four SNPs, rs6913660, rs13219354, rs13211507 and rs9960767, were not polymorphic in samples from the HapMap Japanese in Tokyo (JPT) project. Minor allele frequencies (MAF) of two SNPs, rs6932590 and rs3131296, were under 5%. Because only one marker, rs12807809 in *NRGN*, was a common SNP in HapMap JPT samples (MAF>5%), we focused on this SNP in the present study.

*NRGN* is the human homolog of the neuron-specific rat gene RC3/neurogranin. *NRGN* encodes a postsynaptic protein kinase substrate that binds to calmodulin (CaM) in the absence of calcium [4]. The *NRGN* gene spans 7.3 kb of genomic DNA and contains four exons that transcribe a protein of 78 amino acids [5]. Exons 1 and 2 encode the protein, and exons 3 and 4 contain untranslated sequences. NRGN plays an important role in the Ca^2+^–CaM signaling pathway [6]. A Ca^2+^ influx-induced oxidation of NRGN leads to postsynaptic activation of CaM-dependent protein kinase II (CaMKII) by CaM, which is associated with strengthened *N*-methyl-d-aspartate (NMDA) receptor signaling [7]. Altered NRGN activity may therefore mediate the effects of the NMDA hypofunction implicated in the pathophysiology of schizophrenia.

Many attempts have been made to minimize clinical and genetic heterogeneity in studies of schizophrenia. One strategy for gene discovery uses neurobiological quantitative traits (QT) as intermediate phenotypes rather than the diagnosis of schizophrenia [8], [9]. This strategy has the potential to reduce clinical and genetic heterogeneity by examining intermediate phenotypes that reflect underlying genetic vulnerability better than diagnostic categorization [10]. Structural brain phenotypes are QT that show considerable variation in human populations [11]. A voxel-wise meta-analysis of gray matter (GM) alterations in patients with schizophrenia indicated that they had a reduced GM density in the bilateral insular cortex, anterior cingulate, left parahippocampal gyrus, left middle frontal gyrus, postcentral gyrus, and thalamus and had an increased GM density in the striatal regions relative to the control subjects [12]. A voxel-wise meta-analysis of white matter (WM) alterations in patients with schizophrenia indicated that these patients had a decreased WM volume in the frontal regions and internal capsule relative to control subjects [13]. Heritability estimates indicate a moderate (40–70%) to high (70–95%) genetic influence on brain structure volumes in the frontal and temporal brain regions, such as the middle frontal and the anterior cingulate cortices [11], [14]. Some studies have shown that abnormalities in brain structure are intermediate phenotypes that bridge the gap between the genotype and diagnostic categorization [10], [15], [16]. Our research group has a long-standing interest in the effects of genetic variants on brain structure (i.e., *COMT*, *DISC1*, *PACAP*, *BDNF*, *APOE* and *AKT1*) [17], [18], [19], [20], [21], [22] and on prefrontal activity as measured by near-infrared spectroscopy (NIRS) (*TBP* and *SIGMAR1*) in psychiatric disorders [23], [24]. *NRGN* is expressed exclusively in the brain, especially in the dendritic spines. Reduced NRGN immunoreactivity has been observed in prefrontal areas 9 and 32 of post-mortem schizophrenic brains [25]. To date, no study has investigated the effects of the *NRGN* polymorphism and the genotype-diagnosis interaction on brain morphology at the whole brain level. In this study, we examined the impacts of the *NRGN* polymorphism and the genotype-diagnosis interaction on GM volumes and WM volumes in patients with schizophrenia and in healthy volunteers.

## Materials and Methods

### Ethics statement

Written informed consent was obtained from all subjects after the procedures had been fully explained. This study was carried out in accordance with the World Medical Association's Declaration of Helsinki and approved by the Research Ethical Committee of Osaka University.

### Subjects

Voxel-based morphometry (VBM) analyses were conducted on 99 patients with schizophrenia [52.5% males (52 males and 47 females); mean age ± SD, 38.4±12.9 years] and 263 healthy controls [44.5% males (117 males and 146 females); mean age ± SD, 36.7±11.6 years]. All subjects were biologically unrelated within the second-degree of relationship and of Japanese descent [23], [26]. The subjects were excluded if they had neurological or medical conditions that could potentially affect the central nervous system, such as atypical headache, head trauma with loss of consciousness, chronic lung disease, kidney disease, chronic hepatic disease, thyroid disease, active cancer, cerebrovascular disease, epilepsy, seizures, substance-related disorders or mental retardation. Cases were recruited from the university hospital. Each patient with schizophrenia had been diagnosed by at least two trained psychiatrists according to the criteria of the *Diagnostic and Statistical Manual of Mental Disorders, Fourth Edition* (DSM-IV) based on the Structured Clinical Interview for DSM-IV (SCID). Controls were recruited through local advertisements at Osaka University. Psychiatrically, medically and neurologically healthy controls were evaluated using the non-patient version of the SCID to exclude individuals who had current or past contact with psychiatric services or who had received psychiatric medication. Current symptoms of schizophrenia were evaluated using the positive and negative syndrome scale (PANSS) [27]. Mean age, sex ratio and handedness did not differ significantly between cases and controls (*p*>0.17), while the years of education, estimated premorbid intelligence quotient (IQ) and GM volumes were significantly lower in the patients with schizophrenia than in the controls (*p*<0.001) (Table S1). When the genotype groups were compared, we found no differences in the demographic variables, except for years of education and duration of illness in patients with schizophrenia (Table S1).

### SNP selection and SNP genotyping

We selected rs12807809 in the *NRGN* gene as described in the introduction. This polymorphism is reported as T/C and was previously described in the GWAS [3]. Venous blood was collected from the subjects, and genomic DNA was extracted from whole blood according to standard procedures. The SNP was genotyped using the TaqMan 5′-exonuclease allelic discrimination assay (Assay ID: C__32029000_20, Applied Biosystems, Foster City, California, USA) as previously described [18], [19]. Detailed information on the PCR conditions is available upon request. No deviation from Hardy-Weinberg equilibrium (HWE) in the examined SNP was detected in the patients or in the controls (*p*>0.05).

### Magnetic resonance imaging procedure

All magnetic resonance (MR) studies were performed on a 1.5T GE Sigma EXCITE system. A three-dimensional volumetric acquisition of a T1-weighted gradient echo sequence produced a gapless series of 124 sagittal sections using a spoiled gradient recalled acquisition in the steady state (SPGR) sequence (TE/TR, 4.2/12.6 ms; flip angle, 15°; acquisition matrix, 256×256; 1NEX, FOV, 24×24 cm; slice thickness, 1.4 mm). MR images were processed using optimized VBM in Statistical Parametric Mapping 5 (SPM5) running on MATLAB R2010b (MathWorks, Natick, MA) according to the VBM5.1-Manual (http://dbm.neuro.uni-jena.de/vbm/vbm5-for-spm5/manual/) and as previously described [28], [29]. We screened all scans and found no gross abnormalities, such as infarcts, hemorrhages or brain tumors, in any of the subjects. Each image was visually examined to eliminate images with motion or metal artifacts, and then the anterior commissure-posterior commissure line was adjusted. The normalized segmented images were modulated by multiplication with Jacobian determinants of the spatial normalization function to encode the deformation field for each subject as tissue volume changes in the normal space. Finally, images were smoothed with a 12-mm full-width, half-maximum isotropic Gaussian kernel.

Statistical analyses were performed with SPM8 software (http://www.fil.ion.ucl.ac.uk/spm/software/spm8/). First, we performed whole brain searches to explore the effects of the *NRGN* genotype and the genotype-diagnosis interaction on GM or WM volume in total subjects. Second, we performed separate whole brain searches to explore the effect of the *NRGN* genotype on GM or WM volume in patients with schizophrenia and in controls. The genotype effect on GM or WM volume was assessed statistically using a multiple regression model in SPM8. We contrasted GM or WM volume between the genotype groups (coded as the number of rs12807809 risk T alleles: 0, 1, or 2); GM or WM volumes were correlated with the number of risk T alleles, either positively (CC<CT<TT) or negatively (TT<CT<CC). The genotype-diagnosis interaction on GM or WM volumes was assessed full factorial model with diagnosis as a factor and genotype status as a covariate interacted with the diagnosis in SPM8. Age, sex and years of education were included as covariates of no interest into all analyses to control for confounding variables. Non-sphericity was estimated. These analyses yielded statistical parametric maps {SPM (*t*)} based on a voxel-level height threshold of *p*<0.001 (uncorrected for multiple comparisons). Clusters of more than 100 contiguous voxels were considered in the analyses. Family-wise error (*FWE*) correction was applied for multiple testing to avoid type I errors. The significance level was set at *p*<0.05 (*FWE* corrected). Anatomic localization was performed according to both MNI coordinates and Talairach coordinates, which were obtained from M. Brett's transformations (http://www.mrccbu.cam.ac.uk/Imaging/Common/mnispace.shtml) and presented as Talairach coordinates.

### Statistical analyses

The presence of Hardy-Weinberg equilibrium was examined by the *χ^2^* test for goodness-of-fit using SNPAlyze V5.1.1 Pro software (DYNACOM, Yokohama, Japan). Statistical analyses of demographic variables were performed using PASW Statistics 18.0 software (SPSS Japan Inc., Tokyo, Japan). Differences in clinical characteristics between patients and controls or between genotypes were analyzed using *χ^2^* tests for categorical variables and the Mann-Whitney *U*-test or Kruskal-Wallis test for continuous variables. The significance level for all statistical tests was set at two-tailed *p*<0.05.

## Results

### Effects of the genotype and diagnosis-genotype interaction on GM or WM regions in total subjects

**Figure 1:**
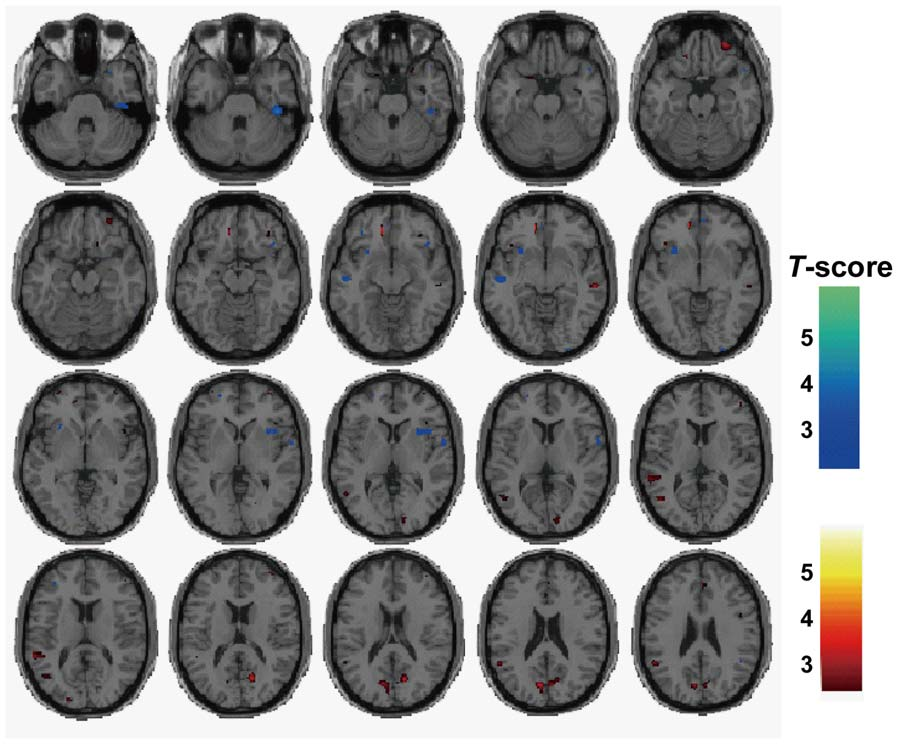
Effects of the risk-T-allele on decreased GM regions and diagnosis-*NRGN* genotype interaction on GM regions. Effects of the risk T allele on decreased GM regions (TT<CT<CC) in total subjects were shown by whinter colormap (blue areas). Diagnosis-*NRGN* genotype interaction on GM regions was shown by hot colormap (red areas). There was no significant effect of the risk T allele on increased GM regions (CC<CT<TT) among the total subjects. Each colormap shows *t* values corresponding to the color in the figure.

First, we investigated the effects of genotype and diagnosis-genotype interaction on GM or WM volumes in the whole brain analyses of total subjects. We found significant effects of the risk T allele on decreased GM volume in the right fusiform gyrus (uncorrected *p*<0.001, Table 1 and blue regions in Figure 1), and on increased WM volume in the inferior parietal lobule among total subjects (uncorrected *p*<0.001, Table 1). We also found significant genotype-diagnosis interaction on GM volume in the left anterior cingulate gyrus and the bilateral precuneus (uncorrected *p*<0.001, Table 1 and red regions in Figure 1). However, the effects of genotype and genotype-diagnosis interaction on these GM or WM regions did not survive after the *FWE*-correction for multiple tests (*FWE*-corrected *p*>0.05). There was no significant effect of the risk T allele on increased GM volumes, the risk T allele on decreased WM volumes, or genotype-diagnosis interaction on WM volume among total subjects (uncorrected *p*>0.001).

**Table 1:** Effects of *NRGN* genotype and genotype-diagnosis interaction on GM and WM volumes in total subjects.

						*p* values		Talairach coordinates		
	Brain regions	R/L	BA	CS	*T*	Uncorrected	*FWE*	*x*	*y*	*z*
**GM**	***NRGN*** ** genotipe-diagnosis interaction**									
	**Limbic Lobe**									
	Anterior Cingulate	L	32	219	4.17	<0.001	0.33	−12	40	−10
	**Occipital Lobe**									
	Precuneus	R	31	118	3.63	<0.001	0.90	15	−64	20
	Precuneus	L	31	165	3.54	<0.001	0.95	−7	−72	25
**GM**	**Total subjects; TT<CT<CC (higher risk<lower risk)**									
	**Temporal Lobe**									
	Fusiform Gyrus	R	20	290	4.28	<0.001	0.25	45	−30	−23
**GM**	**Total subjects; TT>CT>CC (higher risk>lower risk)**									
	no suprathreshold clusters									
**WM**	***NRGN*** ** genotipe-diagnosis interaction**									
	no suprathreshold clusters									
**WM**	**Total subjects; TT<CT<CC (higher risk<lower risk)**									
	no suprathreshold clusters									
**WM**	**Total subjects; TT>CT>CC (higher risk>lower risk)**									
	**Parietal Lobe**									
	Inferior Parietal Lobule	R		616	3.72	<0.001	0.34	44	−41	25

GM: gray matter, WM: white matter, R: right, L: left, BA: Brodmann area, CS: Cluster size, FWE: family-wise error.

### Effect of the risk T allele on decreased GM regions (TT<CT<CC)

**Figure 2:**
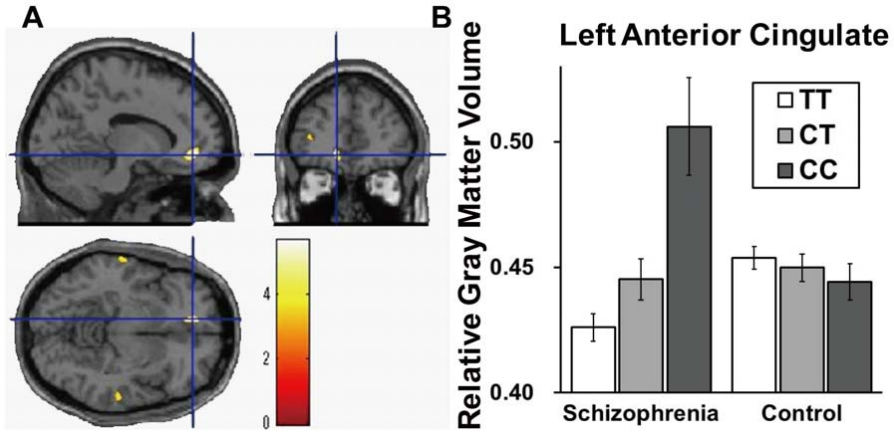
Impact of the *NRGN* genotype on GM volume of left anterior cingulate gyrus in schizophrenia. (**A**) Anatomical localizations are displayed on coronal, sagittal, and axial sections of a normal MRI spatially normalized into the Montreal Neurological Institute template (uncorrected *p*<0.001, cluster size>100). A significant cluster of the genotype effect was in the left anterior cingulate gyrus in the patients with schizophrenia, after controlling for differences in the duration of illness among genotypes. The region is shown as cross-hairline. The color bars show *t* values corresponding to the color in the figure. (**B**) Each column shows relative gray matter volumes extracted from the left anterior cingulate gyrus (Talairach coordinates; −12, 42, −9). We extracted a sphere with a 10 mm volume-of-interest (VOI) radius from the significant region to compare the effects of the genotype in both the patients with schizophrenia and healthy subjects. Error bars represent the standard error.

Second, we separately investigated the effects of genotype on GM or WM volumes in the whole brain analyses of patients with schizophrenia and healthy controls. We found significant effects of the *NRGN* genotype on GM volume in the left anterior cingulate gyrus, the bilateral middle temporal gyrus and the left inferior frontal gyrus among the patients with schizophrenia (uncorrected *p*<0.001, Table 2 and red regions in Figure S1). We found significant effect of the *NRGN* genotype on GM volume in the right fusiform gyrus among the healthy controls (uncorrected *p*<0.001, Table 2 and blue regions in Figure S1). The genotype effect on the left anterior cingulate gyrus (BA32) in the patients with schizophrenia remained significant even after the *FWE*-correction for multiple tests at the whole brain level (*T_94_* = 5.63, *FWE*-corrected *p* = 0.0042, Table 2); genotype effects on other regions did not survive the *FWE*-correction (*FWE*-corrected *p*>0.05). In patients with schizophrenia, the risk T carriers had a smaller GM volume in the left anterior cingulate gyrus than did the non-risk C carriers (Figure 2).

**Table 2:** Effects of *NRGN* genotype on GM volumes in patients with schizophrenia and in healthy controls.

					*p* values		Talairach coordinates		
Brain regions	R/L	BA	CS	*T*	Uncorrected	*FWE*	*x*	*y*	*z*
**SZ; TT<CT<CC (higher risk<lower risk)**									
**Limbic Lobe**									
Anterior Cingulate	L	32	525	5.63	<0.001	**0.0042**	−12	42	−9
**Temporal Lobe**									
Middle Temporal Gyrus	L	21	143	3.87	<0.001	0.80	−66	−19	−5
Middle Temporal Gyrus	R	21	106	3.69	<0.001	0.93	59	−24	−6
**Frontal Lobe**									
Inferior Frontal Gyrus	L	10	102	3.88	<0.001	0.80	−36	45	4
**HC; TT<CT<CC (higher risk<lower risk)**									
**Temporal Lobe**									
Fusiform Gyrus	R	20	334	4.4	<0.001	0.19	45	−31	−23
**SZ; TT>CT>CC (higher risk>lower risk)**									
**Parietal Lobe**									
Precuneus	L	7	182	4	<0.001	0.68	−15	−64	38
**Occipital Lobe**									
Precuneus	R	31	143	3.81	<0.001	0.86	15	−64	19
**HC; TT>CT>CC (higher risk>lower risk)**									
no suprathreshold clusters									

GM: gray matter, R: right, L: left, BA: Brodmann area, CS: Cluster size, FWE: family-wise error, SZ: patients with schizophrenia, HC: healthy controls. Significant results [*p*<0.05 (*FWE* corrected)] are shown as bold face and underline.

Researchers have suggested that the volume reduction of the anterior cingulate cortex (ACC) is associated with the duration of the illness (the length of time the patient has had schizophrenia) [30]. In our samples, the duration of illness differed significantly among the genotype groups in patients with schizophrenia (Table S1). Thus, we corrected for the duration of illness. The genotype effect on the left anterior cingulate gyrus remained significant even after controlling for the duration of illness (*T_93_* = 5.86, *FWE*-corrected *p* = 0.0017).

### Effect of the risk T allele on increased GM regions (CC<CT<TT)

We found significant effects of the *NRGN* genotype on GM volume in the bilateral precuneus among the patients with schizophrenia (uncorrected *p*<0.001, Table 2 and red region in Figure S2); however, the genotype effects on these regions did not survive after the *FWE*-correction for multiple tests (*FWE*-corrected *p*>0.05). There was no significant effect of the *NRGN* genotype on GM volume among the healthy controls (uncorrected *p*>0.001).

### Effects of the risk T allele on WM regions

We found no significant effect of the risk T allele on any decreased WM regions (TT<CT<CC) for either the patients or controls (uncorrected *p*<0.001). On the other hand, we found significant effects of the risk T allele on increased WM region (CC<CT<TT) in the bilateral insula and middle frontal gyrus among the patients with schizophrenia (uncorrected *p*<0.001, Table S2 and red regions in Figure S3). However, the genotype effects on these regions did not survive after the *FWE*-correction (*FWE*-corrected *p*>0.05). There was no significant genotype effect on any increased WM region for the controls (uncorrected *p*<0.001). These findings suggest that *NRGN* may not play a major role in the morphology of WM.

## Discussion

This is the first study to identify brain morphology associated with genome-wide significant risk variants in *NRGN* for schizophrenia at the whole brain level. Genotype-diagnosis interaction on GM volume in the left ACC was found, even though the effect did not survive after the *FWE*-correction. When we separately investigated the effects of the interaction on GM volume of patients with schizophrenia and healthy controls, carrying the risk T allele of rs12807809 was associated with reduced GM volume in the left ACC in patients with schizophrenia. The genotype effect survived a correction for multiple comparisons at the whole brain level. This finding applies to the patients with schizophrenia but not to the healthy controls, and it is present even after controlling for differences in the duration of illness among genotypes. Significant difference on WM volume between genotypes was not observed for any region in patients or controls.

The ACC is a functionally heterogeneous region involved in diverse cognitive processes [30]. The functional diversity of the ACC encompasses executive, attention, social cognitive, affective and skeleton- and viscera-motor functions. Most MRI studies suggest that patients with schizophrenia show reduced GM in the ACC [30]. These reductions extend across the dorsal and rostral divisions of the limbic and paralimbic regions of the ACC. Some studies suggest that relatives of schizophrenia patients also show bilateral reductions in GM volume or thickness in the ACC [31], [32]. Post-mortem findings indicate that these imaging-related changes are accompanied by reductions in neuronal, synaptic, and dendritic density as well as increased afferent input [30]. These findings suggest that the GM differences observed with MRI arise from alterations in both neuronal and non-neuronal tissue compartments.

The GM reductions in the ACC precede the onset of psychosis in some categories of high-risk individuals. Cross-sectional and longitudinal studies suggest that the earliest ACC changes in schizophrenia appear in the rostral paralimbic regions of the ACC prior to the onset of psychosis, extend across the paralimbic regions of the ACC during the transition to a first episode psychosis, and spread to engulf the limbic regions of the ACC with continued illness [30]. The regions of the genotype effect in the present study were the paralimbic regions of the ACC. A mean duration of illness in patients included in this study was 13.0±10.4 years; these patients are considered to have established schizophrenia. As the duration of illness has been related to the degree of reduction of the ACC and because it significantly differed among the genotype groups in our subjects, we ascertained whether the genotype effect in the ACC is affected by variation in the duration of illness. However, the genotype effect in the left ACC was robust even after controlling for the duration of illness. These findings suggest that part of the paralimbic regions of the ACC may be attributed to the effects of the genome-wide supported variant of *NRGN* in patients with schizophrenia, regardless of the duration of illness.

*NRGN* is especially enriched in CA1 pyramidal neurons in the hippocampus [33]. *NRGN* produced severe deficits in hippocampus-dependent tasks in knock-out mice [34], [35]. This evidence suggests that *NRGN* may be important in neurocognitive tasks such as learning and memory and in the morphology and function of the hippocampus. Based on this hypothesis, Donohoe et al. tested the relationship between schizophrenia associated with the *NRGN* variant rs12807809 and cognition in Irish and German case-control samples [36]. They did not find a significant association between the *NRGN* variant and cognition in the samples. Pohlack et al. found that homozygous T carriers had decreased activation of the left hippocampus during contextual fear conditioning but did not find the same result in the hippocampal structure of Caucasian healthy volunteers [37]. We did not find a significant association between the *NRGN* variant and hippocampal volume, consistent with recent study using the ROI approach [37]. These findings suggest that *NRGN* may play an important role in hippocampal activity but not play a major role in the neurocognition of learning and memory or in the morphology of the hippocampus.

There were several limitations to this study. A false-positive association could not be excluded from our study despite the precautions for ethnic matching and corrections for multiple testing. It is necessary to conduct further investigations to confirm our findings in other samples with much larger sample sizes and/or with different ethnicities and/or in relatives with schizophrenia. A false-negative association could not be excluded in our study because we applied a strict correction for multiple comparisons at the whole brain level (*FWE*-corrected *p*<0.05). The regions shown in the Supporting Information (uncorrected *p*<0.001) might be helpful in further studies. It is still unclear whether this genetic variant of the *NRGN* gene is associated with the expression, transcription, splicing or translation of the gene. The lack of a clear association makes it difficult to determine whether our results are directly linked to the *NRGN* polymorphism rs12807809, to other polymorphisms in linkage disequilibrium with this variant, or to interaction between this genetic variant of the *NRGN* and other polymorphism. As with other risk variants for schizophrenia, clarifying the biological role of this variant through *in vitro* and *in vivo* studies is important to improve the understanding of the pathophysiology of schizophrenia. In addition, an extensive search for other functional variants at this locus is needed to determine whether rs12807809 is the most strongly associated variant for schizophrenia in this gene.

In conclusion, we found that a genome-wide supported variant of *NRGN* may be associated with brain morphological vulnerability of the left ACC in patients with schizophrenia. Abnormalities in ACC may partly explain the disturbances in cognitive and emotional integration in patients with schizophrenia. Further research will be required to clarify the function of the risk *NRGN* variant on the pathophysiology of schizophrenia.

## Supporting Information

Figure S1 **Effect of risk-T-allele on decreased GM regions in patients with schizophrenia and in healthy controls.** Effect of the risk T allele on decreased GM regions (TT<CT<CC) in the patients with schizophrenia was shown by hot colormap (red areas), while effect of the T allele on decreased GM regions in the healthy controls was shown by winter colormap (blue areas).
(TIF)

Figure S2 **Effect of the risk-T-allele on increased GM regions in the patients with schizophrenia.** Effect of the risk T allele on increased GM regions (CC<CT<TT) in the patients with schizophrenia was shown by hot colormap (red areas). There was no significant effect of the *NRGN* genotype on GM volume among the healthy controls.
(TIF)

Figure S3 **Effect of the risk-T-allele on increased WM regions in the patients with schizophrenia.** Effect of the risk T allele on increased WM regions (CC<CT<TT) in the patients with schizophrenia was shown by hot colormap (red areas). There was no significant effect of the *NRGN* genotype on WM volume among the healthy controls.
(TIF)

Table S1 **Demographic information for patients with schizophrenia and healthy controls included in the VBM analysis.**
(DOC)

Table S2 **Effects of the** ***NRGN*** **genotype on WM volumes in patients with schizophrenia and healthy controls.**
(DOC)

## References

[1] Sullivan PF, Kendler KS, Neale MC (2003). Schizophrenia as a complex trait: evidence from a meta-analysis of twin studies.. Arch Gen Psychiatry.

[2] Sun J, Kuo PH, Riley BP, Kendler KS, Zhao Z (2008). Candidate genes for schizophrenia: a survey of association studies and gene ranking.. Am J Med Genet B Neuropsychiatr Genet.

[3] Stefansson H, Ophoff RA, Steinberg S, Andreassen OA, Cichon S (2009). Common variants conferring risk of schizophrenia.. Nature.

[4] Baudier J, Deloulme JC, Van Dorsselaer A, Black D, Matthes HW (1991). Purification and characterization of a brain-specific protein kinase C substrate, neurogranin (p17). Identification of a consensus amino acid sequence between neurogranin and neuromodulin (GAP43) that corresponds to the protein kinase C phosphorylation site and the calmodulin-binding domain.. J Biol Chem.

[5] Martinez de Arrieta C, Perez Jurado L, Bernal J, Coloma A (1997). Structure, organization, and chromosomal mapping of the human neurogranin gene (NRGN).. Genomics.

[6] Hayashi Y (2009). Long-term potentiation: two pathways meet at neurogranin.. EMBO J.

[7] Li J, Pak JH, Huang FL, Huang KP (1999). N-methyl-D-aspartate induces neurogranin/RC3 oxidation in rat brain slices.. J Biol Chem.

[8] Meyer-Lindenberg A, Weinberger DR (2006). Intermediate phenotypes and genetic mechanisms of psychiatric disorders.. Nat Rev Neurosci.

[9] Tan HY, Callicott JH, Weinberger DR (2008). Intermediate phenotypes in schizophrenia genetics redux: is it a no brainer?. Mol Psychiatry.

[10] Potkin SG, Turner JA, Guffanti G, Lakatos A, Torri F (2009). Genome-wide strategies for discovering genetic influences on cognition and cognitive disorders: methodological considerations.. Cogn Neuropsychiatry.

[11] Kaymaz N, van Os J (2009). Heritability of structural brain traits an endophenotype approach to deconstruct schizophrenia.. Int Rev Neurobiol.

[12] Glahn DC, Laird AR, Ellison-Wright I, Thelen SM, Robinson JL (2008). Meta-analysis of gray matter anomalies in schizophrenia: application of anatomic likelihood estimation and network analysis.. Biol Psychiatry.

[13] Di X, Chan RC, Gong QY (2009). White matter reduction in patients with schizophrenia as revealed by voxel-based morphometry: an activation likelihood estimation meta-analysis.. Prog Neuropsychopharmacol Biol Psychiatry.

[14] Rijsdijsk FV, Viding E, De Brito S, Forgiarini M, Mechelli A (2010). Heritable variations in gray matter concentration as a potential endophenotype for psychopathic traits.. Arch Gen Psychiatry.

[15] Prasad KM, Keshavan MS (2008). Structural cerebral variations as useful endophenotypes in schizophrenia: do they help construct “extended endophenotypes”?. Schizophr Bull.

[16] Goldman AL, Pezawas L, Mattay VS, Fischl B, Verchinski BA (2009). Widespread reductions of cortical thickness in schizophrenia and spectrum disorders and evidence of heritability.. Arch Gen Psychiatry.

[17] Ohnishi T, Hashimoto R, Mori T, Nemoto K, Moriguchi Y (2006). The association between the Val158Met polymorphism of the catechol-O-methyl transferase gene and morphological abnormalities of the brain in chronic schizophrenia.. Brain.

[18] Hashimoto R, Hashimoto H, Shintani N, Chiba S, Hattori S (2007). Pituitary adenylate cyclase-activating polypeptide is associated with schizophrenia.. Mol Psychiatry.

[19] Hashimoto R, Numakawa T, Ohnishi T, Kumamaru E, Yagasaki Y (2006). Impact of the DISC1 Ser704Cys polymorphism on risk for major depression, brain morphology and ERK signaling.. Hum Mol Genet.

[20] Hashimoto R, Moriguchi Y, Yamashita F, Mori T, Nemoto K (2008). Dose-dependent effect of the Val66Met polymorphism of the brain-derived neurotrophic factor gene on memory-related hippocampal activity.. Neurosci Res.

[21] Ohi K, Hashimoto R, Yasuda Y, Fukumoto M, Nemoto K (2011). The AKT1 gene is associated with attention and brain morphology in schizophrenia.. World J Biol Psychiatry.

[22] Hashimoto R, Hirata Y, Asada T, Yamashita F, Nemoto K (2009). Effect of the brain-derived neurotrophic factor and the apolipoprotein E polymorphisms on disease progression in preclinical Alzheimer's disease.. Genes Brain Behav.

[23] Ohi K, Hashimoto R, Yasuda Y, Kiribayashi M, Iike N (2009). TATA box-binding protein gene is associated with risk for schizophrenia, age at onset and prefrontal function.. Genes Brain Behav.

[24] Ohi K, Hashimoto R, Yasuda Y, Fukumoto M, Yamamori H (2011). The SIGMAR1 gene is associated with a risk of schizophrenia and activation of the prefrontal cortex.. Prog Neuropsychopharmacol Biol Psychiatry.

[25] Broadbelt K, Ramprasaud A, Jones LB (2006). Evidence of altered neurogranin immunoreactivity in areas 9 and 32 of schizophrenic prefrontal cortex.. Schizophr Res.

[26] Hashimoto R, Ohi K, Yasuda Y, Fukumoto M, Iwase M (2010). The impact of a genome-wide supported psychosis variant in the ZNF804A gene on memory function in schizophrenia.. Am J Med Genet B Neuropsychiatr Genet.

[27] Lindenmayer JP, Bernstein-Hyman R, Grochowski S (1994). A new five factor model of schizophrenia.. Psychiatric Quarterly.

[28] Good CD, Johnsrude IS, Ashburner J, Henson RN, Friston KJ (2001). A voxel-based morphometric study of ageing in 465 normal adult human brains.. Neuroimage.

[29] Ashburner J, Friston KJ (2000). Voxel-based morphometry–the methods.. Neuroimage.

[30] Fornito A, Yucel M, Dean B, Wood SJ, Pantelis C (2009). Anatomical abnormalities of the anterior cingulate cortex in schizophrenia: bridging the gap between neuroimaging and neuropathology.. Schizophr Bull.

[31] Goghari VM, Rehm K, Carter CS, MacDonald AW (2007). Regionally specific cortical thinning and gray matter abnormalities in the healthy relatives of schizophrenia patients.. Cereb Cortex.

[32] Bhojraj TS, Sweeney JA, Prasad KM, Eack SM, Francis AN (2011). Gray matter loss in young relatives at risk for schizophrenia: relation with prodromal psychopathology.. Neuroimage.

[33] Huang FL, Huang KP, Boucheron C (2007). Long-term enrichment enhances the cognitive behavior of the aging neurogranin null mice without affecting their hippocampal LTP.. Learn Mem.

[34] Pak JH, Huang FL, Li J, Balschun D, Reymann KG (2000). Involvement of neurogranin in the modulation of calcium/calmodulin-dependent protein kinase II, synaptic plasticity, and spatial learning: a study with knockout mice.. Proc Natl Acad Sci U S A.

[35] Huang KP, Huang FL, Jager T, Li J, Reymann KG (2004). Neurogranin/RC3 enhances long-term potentiation and learning by promoting calcium-mediated signaling.. J Neurosci.

[36] Donohoe G, Walters J, Morris DW, Da Costa A, Rose E (2011). A neuropsychological investigation of the genome wide associated schizophrenia risk variant NRGN rs12807809.. Schizophr Res.

[37] Pohlack ST, Nees F, Ruttorf M, Witt SH, Nieratschker V (2011). Risk variant for schizophrenia in the neurogranin gene impacts on hippocampus activation during contextual fear conditioning.. Mol Psychiatry.

