# Supplementary figures and images for: Impact of the Genome Wide Supported NRGN Gene on Anterior Cingulate Morphology in Schizophrenia
Source: PLoS One. 2012 Jan 12;7(1):e29780. doi: 10.1371/journal.pone.0029780 (PMC3257237; doi:10.1371/journal.pone.0029780)

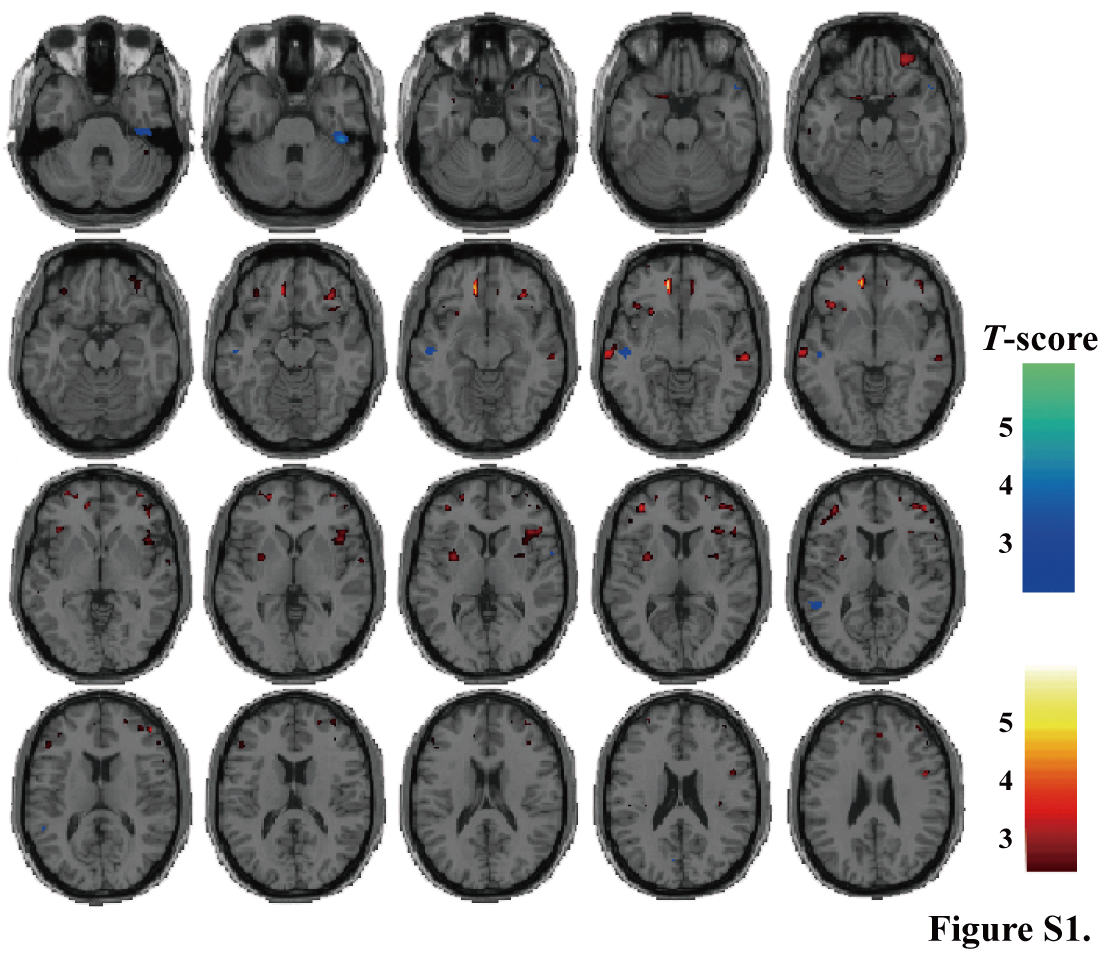

Supplement: Figure S1 — Effect of risk-T-allele on decreased GM regions in patients with schizophrenia and in healthy controls. Effect of the risk T allele on decreased GM regions (TT<CT<CC) in the patients with schizophrenia was shown by hot colormap (red areas), while effect of the T allele on decreased GM regions in the healthy controls was shown by winter colormap (blue areas). (TIF) [file pone.0029780.s001.tif]

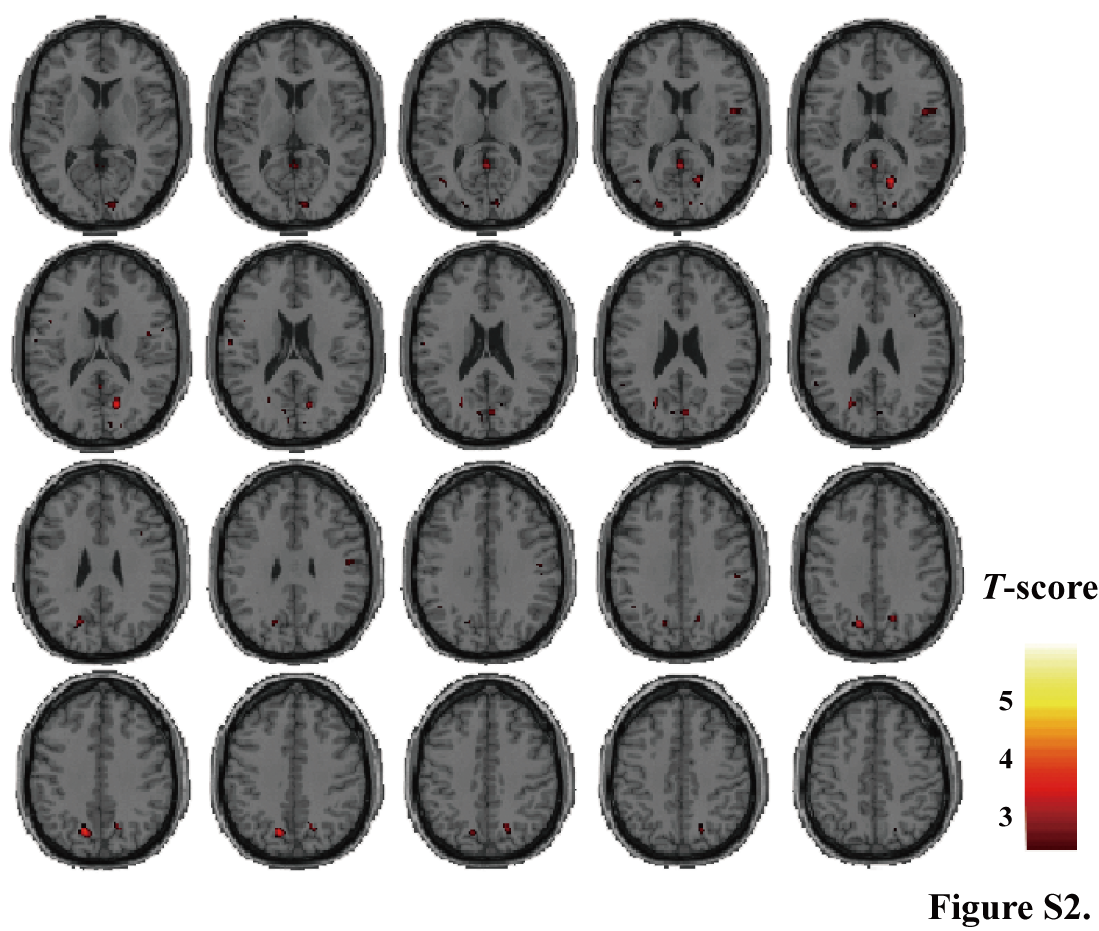

Supplement: Figure S2 — Effect of the risk-T-allele on increased GM regions in the patients with schizophrenia. Effect of the risk T allele on increased GM regions (CC<CT<TT) in the patients with schizophrenia was shown by hot colormap (red areas). There was no significant effect of the NRGN genotype on GM volume among the healthy controls. (TIF) [file pone.0029780.s002.tif]

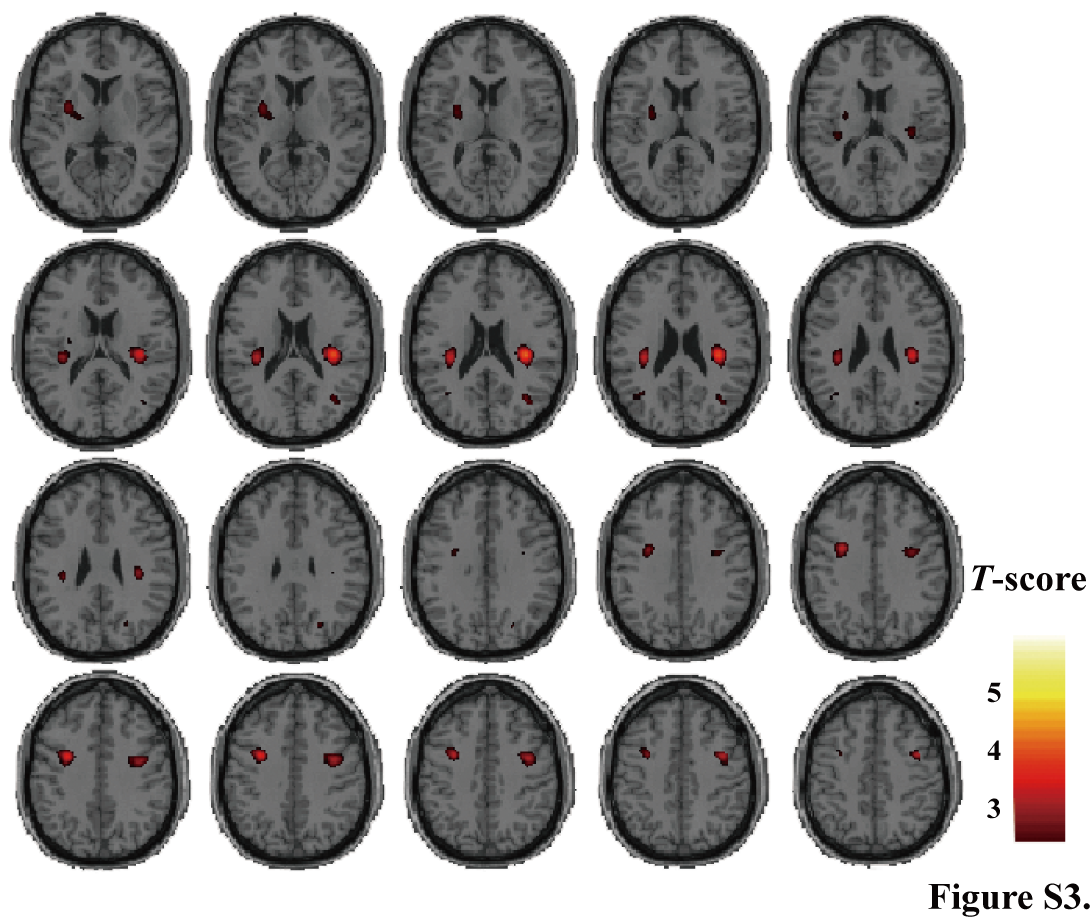

Supplement: Figure S3 — Effect of the risk-T-allele on increased WM regions in the patients with schizophrenia. Effect of the risk T allele on increased WM regions (CC<CT<TT) in the patients with schizophrenia was shown by hot colormap (red areas). There was no significant effect of the NRGN genotype on WM volume among the healthy controls. (TIF) [file pone.0029780.s003.tif]
