# Supplementary material for: Impact of the Genome Wide Supported NRGN Gene on Anterior Cingulate Morphology in Schizophrenia
Source: PLoS One. 2012 Jan 12;7(1):e29780. doi: 10.1371/journal.pone.0029780 (PMC3257237; doi:10.1371/journal.pone.0029780)
Supplement: Table S1 — Demographic information for patients with schizophrenia and healthy controls included in the VBM analysis. (DOC) [file pone.0029780.s004.doc]

**Table S1.** Demographic information for patients with schizophrenia and healthy controls included in the VBM analysis.

|  | **Schizophrenia (*N* = 99)** | |  |  |  | **Control (*N* = 263)** | |  |  |  | **Group difference** |
| --- | --- | --- | --- | --- | --- | --- | --- | --- | --- | --- | --- |
|  | **TT** | **CT** | **CC** | ***p* values (*H*)** |  | **TT** | **CT** | **CC** | ***p* values (*H*)** |  | ***p* values (*z*)** |
| **Variables** | **(*N* = 59)** | **(*N* = 36)** | **(*N* = 4)** |  |  | **(*N* = 148)** | **(*N* = 94)** | **(*N* = 21)** |  |  |  |
| Age (years) | 36.3 ± 13.0 | 41.2 ± 11.3 | 44.8 ± 22.2 | 0.07 (5.27) |  | 36.3 ± 11.3 | 37.6 ± 12.0 | 35.4 ± 11.5 | 0.60 (0.80) |  | 0.35 (0.95) |
| Sex (male/female) | 29/30 | 20/16 | 3/1 | 0.55 (1.21)^a^ |  | 65/83 | 44/50 | 8/13 | 0.75 (0.57)^a^ |  | 0.17 (1.87)^a^ |
| Education (years) | 13.3 ± 2.2 | 14.9 ± 2.4 | 13.5 ± 1.7 | **0.016 (8.33)** |  | 15.2 ± 2.2 | 14.9 ± 2.4 | 15.3 ± 2.6 | 0.57 (1.13) |  | **<0.001 (-4.18)** |
| Estimated premorbid IQ | 102.0 ± 10.1 | 100.1 ± 10.7 | 101.0 ± 1.7 | 0.68 (0.78) |  | 106.7 ± 8.3 | 107.5 ± 8.1 | 108.4 ± 7.0 | 0.83 (0.37) |  | **<0.001 (-4.96)** |
| Handedness (rt./lt./bil.) | 58/1/0 | 34/2/0 | 3/1/0 | 0.06 (5.58)^a^ |  | 140/7/0 | 87/7/0 | 20/1/0 | 0.81 (1.59)^a^ |  | 0.68 (0.79)^a^ |
| Gray matter volume (mm3) | 679.1 ± 75.7 | 670.3 ± 81.7 | 675.0 ± 97.6 | 0.78 (0.49) |  | 707.1 ± 79.0 | 695.1 ± 71.5 | 714.1 ± 80.6 | 0.59 (1.06) |  | **0.003 (-2.95)** |
| CPZeq (mg/day) | 636.1 ± 585.8 | 555.4 ± 461.6 | 307.5 ± 149.1 | 0.66 (0.84) |  | - | - | - | - |  | - |
| Age at onset (years) | 26.5 ± 11.8 | 22.9 ± 7.8 | 30.8 ± 15.0 | 0.29 (2.48) |  | - | - | - | - |  | - |
| Duration of illness (years) | 9.8 ± 7.8 | 18.2 ± 11.7 | 14.0 ± 14.9 | **<0.001 (13.01)** |  | - | - | - | - |  | - |
| PANSS positive symptoms | 18.7 ± 5.9 | 18.5 ± 6.3 | 21.5 ± 3.9 | 0.52 (1.33) |  | - | - | - | - |  | - |
| PANSS negative symptoms | 19.5 ± 6.4 | 18.2 ± 6.7 | 21.0 ± 2.2 | 0.39 (1.90) |  | - | - | - | - |  | - |
| PANSS general psychopathology | 40.2 ± 11.0 | 39.1 ± 11.6 | 39.5 ± 7.7 | 0.94 (0.13) |  | - | - | - | - |  | - |

PANSS: Positive and Negative Syndrome Scale, CPZ-eq: chlorpromazine equivalent of total antipsychotics. Means ± SD and *p* values are shown. Significant p values are shown as bold face and underline. ^a^*χ^2^* test. Complete demographic information was not obtained for all subjects (estimated premorbid IQ in patients: TT, N = 58: CT, N = 33: CC, N = 3, estimated premorbid IQ in controls: CT, N = 93; PANSS: TT, N = 58: CT, N = 35).
