# Supplementary material for: Impact of the Genome Wide Supported NRGN Gene on Anterior Cingulate Morphology in Schizophrenia
Source: PLoS One. 2012 Jan 12;7(1):e29780. doi: 10.1371/journal.pone.0029780 (PMC3257237; doi:10.1371/journal.pone.0029780)
Supplement: Table S2 — Effects of the NRGN genotype on WM volumes in patients with schizophrenia and healthy controls. (DOC) [file pone.0029780.s005.doc]

**Table S2.** Effects of the *NRGN* genotype on WM volumes in patients with schizophrenia and healthy controls.

|  |  |  |  | *p* values |  | Talairach coordinates | | |
| --- | --- | --- | --- | --- | --- | --- | --- | --- |
| Brain regions | R/L | CS | *T* | Uncorrected | *FWE* | *x* | *y* | *z* |
| **SZ; TT < CT < CC (higher risk < lower risk)** | | | |  |  |  |  |  |
| no suprathreshold clusters |  |  |  |  |  |  |  |  |
| **HC; TT < CT < CC (higher risk < lower risk)** | | | | |  |  |  |  |
| no suprathreshold clusters |  |  |  |  |  |  |  |  |
| **SZ; CC < CT < TT (lower risk < higher risk)** | | | | |  |  |  |  |
| Insula | R | 1044 | 4.26 | <0.001 | 0.11 | 36 | -22 | 21 |
| Insula | L | 289 | 3.57 | <0.001 | 0.57 | -33 | -24 | 21 |
| Middle Frontal Gyrus | L | 395 | 3.78 | <0.001 | 0.38 | -29 | 3 | 36 |
| Middle Frontal Gyrus | R | 206 | 3.43 | <0.001 | 0.71 | 39 | 4 | 41 |
| **HC; CC < CT < TT (lower risk < higher risk)** | | | | |  |  |  |  |
| no suprathreshold clusters |  |  |  |  |  |  |  |  |

WM: white matter, R: right, L: left, CS: Cluster size, FWE: family-wise error, SZ: patients with schizophrenia, HC: healthy controls.
